# Endophytic Bacteria Associated with *Origanum* *heracleoticum* L. (Lamiaceae) Seeds

**DOI:** 10.3390/microorganisms10102086

**Published:** 2022-10-21

**Authors:** Giulia Semenzato, Teresa Faddetta, Sara Falsini, Sara Del Duca, Antonia Esposito, Anna Padula, Claudia Greco, Nadia Mucci, Marco Zaccaroni, Anna Maria Puglia, Alessio Papini, Renato Fani

**Affiliations:** 1 Department of Biology, University of Florence, Via Madonna del Piano 6, Sesto Fiorentino, 50019 Florence, Italy; 2 Department of Biological, Chemical and Pharmaceutical Sciences and Technologies-STEBICEF, University of Palermo, Viale delle Scienze Ed. 17, 90128 Palermo, Italy; 3 Unit for Conservation Genetics (BIO-CGE), Institute for Environmental Protection and Research, Via Ca’ Fornacetta, 9, Ozzano dell’Emilia, 40064 Bologna, Italy

**Keywords:** medicinal plants, microbiome, seed-associated endophytes, essential oil, antimicrobial compounds, phytobiome

## Abstract

Seed-associated microbiota are believed to play a crucial role in seed germination, seedling establishment, and plant growth and fitness stimulation, due to the vertical transmission of a core microbiota from seeds to the next generations. It might be hypothesized that medicinal and aromatic plants could use the seeds as vectors to vertically transfer beneficial endophytes, providing plants with metabolic pathways that could influence phytochemicals production. Here, we investigated the localization, the structure and the composition of the bacterial endophytic population that resides in *Origanum heracleoticum* L. seeds. Endocellular bacteria, surrounded by a wall, were localized close to the aleurone layer when using light and transmission electron microscopy. From surface-sterilized seeds, cultivable endophytes were isolated and characterized through RAPD analysis and 16S RNA gene sequencing, which revealed the existence of a high degree of biodiversity at the strain level and the predominance of the genus *Pseudomonas*. Most of the isolates grew in the presence of six selected antibiotics and were able to inhibit the growth of clinical and environmental strains that belong to the *Burkholderia cepacia* complex. The endophytes production of antimicrobial compounds could suggest their involvement in plant secondary metabolites production and might pave the way to endophytes exploitation in the pharmaceutical field.

## 1. Introduction

Plants are complex and dynamic organisms that harbor an intricate network of microorganisms, constituting the plant microbiota. Those microbes that are able to colonize the internal tissues of the plant, without causing any visible adverse effects on their host, are referred to as endophytes [1,2]. The majority of bacterial endophytes originate from the rhizosphere, due to the cross-talk that exists between the bacteria and the host plant, whose production of root exudates attracts soil-borne microorganisms [3]. However, bacteria can also be recruited from stem and leaf surfaces and the reproductive organs, such as flowers, fruits, and seeds [4].

Plant endophytes can benefit from large amounts of nutrients, low competition, and protection against environmental and biotic stress [5]. On the other side, several endophytes can improve plant growth and health, protecting their host from phytopathogens through the synthesis of secondary metabolites and/or the stimulation of plant defense responses, or increasing tolerance to abiotic stress, facilitating nutrient acquisition, and secreting phytohormones [3]. Since each plant microenvironment allows the survival of those microorganisms that possess the appropriate metabolic attributes to colonize and inhabit that specific plant organ, different microbial communities can be found across the plant [1].

One of the most crucial stages of a plant’s life history is the seed-to-seedling transition. Germinating seeds are particularly sensitive to drought, granivores, and microbial pathogens and even after their germination, seedlings are constantly exposed to threats that interfere with their establishment. For these reasons, the microbial interactions that take place before and during these phases are pivotal in defining plant development and dynamics [6].

Bacterial endophytes, mostly Proteobacteria, Actinobacteria, Bacteroidetes and Firmicutes, are supposed to be present in seeds of all plant species [4,7]. However, only specific microorganisms can colonize the internal desiccated tissues of the seed [1]. During seed maturation, starch accumulation and drying allow the establishment of those bacteria that can survive in the presence of high osmotic pressure; indeed, the composition of the seed-inhabiting bacterial populations can shift along seed developmental stages [8].

Bacterial seed endophytes mainly originate from the floral microbiota (arising from atmospheric deposition, rainfall, or pollinators) or from the rhizosphere, reaching the flower through the xylemic vessels [6]. Beyond these two transmission routes, external pathways are also involved in the structuring of the seed-associated microbiome [9,10]. After germination, seeds imbibe water and start to secrete various primary and secondary metabolites that attract bacteria from the surrounding environment [7]. These second colonizers compete with the already established microbiota, which may have an ecological advantage due to their proximity to the resources [5,9]. The final composition of the seed microbiota can impact seed quality and also plant fitness, affecting seed viability, germination and seedling survival [1,5].

From an ecological viewpoint, seed microbiota represents “both an endpoint for community assembly within the seed, and also a starting point for community assembly of the new seedling microbiome” [9], to which they can provide benefits [11,12]. It has been suggested that plants can transmit a core microbiota across generations, using seeds as vectors [3]. The existence of a core seed microbiota was observed for cucurbit and rice species [11,13], and also for maize seeds, in which endophytes diversity turned out to be conserved to a certain extent across evolution, human selection and cross-continental migration [14]. Moreover, vertical transmission was demonstrated by Mitter et al. (2017), who were able to modulate the seed and the offspring generation microbiome by performing a spray inoculation in flowers, obtaining seeds and germinated plants colonized by the inoculant strain [15]. Seed endophytes are believed to be able to colonize the seedling, and subsequentially the adult plant, (i) by exiting the seed and then entering the plant from the roots or the above-ground surface or (ii) by remaining inside the seed to be maintained as endophytes as the plant grows [7].

The process of vertical transmission may allow plants to transfer endophytic bacteria with beneficial characteristics to their offspring [7]. As founders of the plant microbiota, seed-borne endophytes might increase seedling fitness, giving the host an advantage over other plant communities when competing for the same ecological niche [13]. Other beneficial activities reported for seed-associated endophytes involve nutrient acquisition and mobilization, protection from pathogens and modulation of plant metabolism [5]. As they possess plant growth-promoting and biocontrol activities, their employment in the agricultural field, such as in biofertilization and bioremediation processes, should be supported [7]. At the same time, their involvement in the plant secondary metabolism should be further investigated, especially in medicinal and aromatic plants, for which it is presumed that the plant-associated endophytic community is directly or indirectly involved in the synthesis of bioactive compounds [16,17]. Indeed, medicinal plants and their endophytes contribute to more than 80% of the available natural drugs, and endophytic microorganisms are reservoirs of novel secondary metabolites with anti-arthritic, antimicrobial, anticancer, antidiabetic, anti-insect, and immunosuppressant activities [18,19].

Focusing on the ever-increasing emergence of multidrug-resistant human pathogens, medicinal plants with antibacterial properties (and some of their associated endophytic bacteria) could represent a natural source of bioactive molecules to be exploited in order to overcome the antibiotic resistance issue [20]. Among others, *Origanum vulgare* L. and its subspecies are Mediterranean medicinal and aromatic plants that are widely studied for their phytotherapeutic potential. Antibacterial and antifungal activities have been intensively explored for *O. vulgare* essential oil, but also anti-inflammatory, antioxidant, cytotoxic and beneficial effects for skin disorders have been reported [21].

Given the fundamental role played by seed-associated endophytes in enhancing plant growth and fitness and considering their putative involvement in plant secondary metabolism [22], it might be suggested that medicinal and aromatic plants could vertically transfer beneficial endophytes to the next generation of plants, providing them with metabolic features that could influence essential oil production.

In this work, the culturable bacterial endophytes that reside within the seeds of *O. heracleoticum* L., synonym of *Origanum vulgare* L. subsp. *viridulum* (Martrin-Donos) Nyman [23,24], were isolated and characterized by a combination of phenotypic and molecular approaches to explore their antimicrobial potential and to identify some of the bacterial features that could be involved in the colonization of the seed microenvironment.

## 2. Materials and Methods

### 2.1. Light Microscopy and Transmission Electron Microscopy

Fifteen seeds of *O. heracleoticum* were cut in two halves and prefixed in 1.25% glutaraldehyde at 4° C in 0.1 M phosphate buffer (pH 6.8) for 10 h, then post-fixed in 1% OsO_4_ in the same buffer for 1 hour. This was followed by dehydration in an ethanol series, with a final propylene oxide step. The samples were embedded in Spurr’s epoxy resin [25]. Seeds embedded in Spurr’s epoxy resin were cross sectioned with a glass knife to obtain semithin sections (1–3 μm), then stained with toluidine blue. The sections were observed and photographed with a Leitz DM RB light microscope. Samples embedded in Spurr’s resin were also cut with a diamond knife to create sections that were approximately 80 nm thick and put on copper grids. The sections were stained with uranyl acetate and lead citrate, and then examined with a Philips EM201 TEM, operating at 80 kV.

### 2.2. Cultivable Endophytic Bacteria Isolation and Growth Conditions

*O. heracleoticum* seeds (100 mg) were surface-sterilized following the procedures described by Faddetta et al. (2021) with modifications [26]. In particular, immersion in sterile distilled water for 1 min was followed by subsequent immersion into 70% (*v*/*v*) ethanol (30 sec), 1.25% (*v*/*v*) sodium hypochlorite solution (30 sec), 70% (*v*/*v*) ethanol (30 sec) and finally, two rinses in sterile distilled water. To confirm that the sterilization process was successful, 1 mL of the water used for the final washing of surface-sterilized seeds was plated on tryptic soy agar (TSA) medium and examined for microbial growth after incubation at 30 °C for 7 days. The surface-sterilized seeds were immersed in sterile distilled water for 1 h at room temperature. Then, to obtain a homogenate containing endophytes, seeds were grounded using a Potter–Elvehjem Tissue Grinder in 2 mL of phosphate saline buffer (Corning PBS) and finally shaken at 150 rpm for 1 h. Several aliquots (100 μL) were then plated on TSA medium. The plates were incubated at 30 °C for 7 days. The bacterial colonies obtained were selected by phenotypic criteria (pigmentation and morphology) and repeatedly incubated on agar media to obtain pure cultures. The isolates are referred to as OR, followed by a number.

### 2.3. Random Amplified Polymorphic DNA (RAPD) Analysis

RAPD analysis was performed, as described in the work of Castronovo et al. (2020) [27]. For each pure culture, a single isolated colony was resuspended in 20 μL of sterile distilled H_2_O and cell lysates were prepared by thermal lysis (95 °C for 10 min), followed by cooling on ice for 5 min. RAPD profiles were obtained as follows: the reaction was performed in a 25 μL volume with 1× DreamTaq Buffer, 200 μM dNTPs, 500 ng of primer 1253 (5′-GTTTCCGCCC-3′) or primer AP12 (5′-CGGCCCCTGC-3′), 1 U of DreamTaq DNA Polymerase (Thermo Scientific, Waltham, MA, USA), and 2 μL of thermal lysate used as template. The reaction mix was incubated in a Bio-Rad T100 thermal cycler at 90 °C for 1 min and 95 °C for 95 s, followed by 45 cycles of 95 °C for 30 s, 36 °C for 1 min, and 75 °C for 2 min; finally, the mixtures were incubated at 75 °C for 10 min and 60 °C for 10 min. Amplicons were visualized through 2% *w*/*v* agarose gel electrophoresis and assigned to haplotype groups, by comparing the fingerprint pattern of each RAPD product for the presence/absence of bands.

### 2.4. 16S rRNA Gene Amplification and Sequencing and ARDRA Analysis

For each recognized RAPD haplotype, the 16S rRNA coding gene sequence was used for taxonomic attribution. Amplification of 16S rRNA gene was performed in a total volume of 20 μL, containing 1× DreamTaq Buffer, 250 μM dNTPs, 0.6 μM primers P0 (5′-GAGAGTTTGATCCTGGCTCAG-3′) and P6 (5′-CTACGGCTACCTTGTTACGA-3′), 1 U of Dream Taq DNA Polymerase (Thermo Scientific), and 1 μL of thermal lysate used as template. The reaction mix was incubated in a thermal cycler (Bio-Rad T100) at 95 °C for 30 s, followed by 30 cycles of 30 s at 95 °C, 30 s at 50 °C, and 1 min at 72 °C, with a final extension step at 72 °C for 10 min. Amplicons were analyzed through 0.8% *w*/*v* agarose gel electrophoresis. A 4 μL aliquot of each PRC product (about 1.5 μg of DNA) was treated with 5U of the restriction enzyme *Alu*I, in a total volume of 30 μL, at 37 °C for 3 h (Amplified Ribosomal DNA Restriction Analysis, ARDRA). The reaction products were visualized through 2.5% *w*/*v* agarose gel electrophoresis and the profiles obtained were compared to identify strains that belonged to the same species [28]. A representative for each profile was chosen to proceed with the gene sequencing.

PCR products were purified with the ExoSAP-IT *Express* PCR Product Cleanup Kit (AppliedBiosystems, Waltham, MA, USA) and then sequenced using the BigDye Terminator v.31 Cycle Sequencing Kit (AppliedBiosystems). Sequences were displayed using the software Chromas 2.6.6 (Technolysium Pty Ltd, South Brisbane, QLD, Australia). Detected sequences were compared with those deposited in the NCBI database through the Basic Local Alignment Search Tool (BLAST) [29]. Each sequence was submitted to GenBank, under the accession numbers OP522403 to OP522415.

### 2.5. Phylogenetic Tree Construction

The 16S rRNA gene sequences were aligned with type-strain sequences downloaded from the Ribosomal Database Project (RDP) [30], using MEGA XI [31]. The alignment was then used to build a phylogenetic tree through MEGA XI for each genus, applying the neighbor-joining algorithm with a 1000-bootstrap resampling, using the Kimura 2-parameter model. 16S RNA gene sequences obtained from *Echinacea purpurea* seed-associated endophytes [32] were also used for the construction of some of the phylogenetic trees.

### 2.6. Antibiotic Resistance Tests

Evaluation of antibiotic resistance was performed by streaking each strain on TSA medium that contained increasing concentrations of different antibiotics, according to the work of Mengoni et al. (2014) [33]. A colony of each strain was suspended in 100 μL saline solution (0.9% *w*/*v* NaCl), streaked on TSA medium supplemented with antibiotics and then incubated at 30 °C for 48 h. The selected antibiotics were tested at the following concentrations (μg/mL): streptomycin, kanamycin and ciprofloxacin (0.5–1–2.5–5–10–50); tetracycline (0.5–1.25–2.5–5–12.5–25); chloramphenicol (1–2.5–5–10–25-50); rifampicin (5–10–25–50–100). The different growth levels were indicated as complete (4), strong (3), weak (2), very weak (1), and absence of growth (0), and MIC (minimal inhibitory concentration) values were identified.

### 2.7. Cross-Streaking Test against Burkholderia cepacia Complex Strains

Seed endophytes’ antimicrobial activity was evaluated through cross-streaking against eleven selected strains that belonged to the *Burkholderia cepacia* complex (Bcc), MDR bacteria that are able to resist different antibiotic classes. The strains were previously isolated from cystic fibrosis (CF) patients and from the environment (Table 1) [34,35]. Tester endophytic strains were streaked across one half of a TSA plate and grown at 30 °C for 48 h to allow the production of antimicrobial compounds. Target Bcc strains were then streaked perpendicularly to the tester strain and plates were incubated at 30 °C for a further 48 h. Target strains were also grown at 30 °C for 48 h in the absence of the tester, as a growth control. The antagonistic effect was evaluated as the absence or reduction in the target strain growth compared to the control. The degrees of inhibition were indicated as complete (3, red), strong (2, orange), weak (1, salmon), and absence (0, white) of inhibition [36].

### 2.8. Genome Sequencing

Nanopore sequencing was performed with a PCR-free approach, following the native barcoding genomic DNA protocol provided by Oxford Nanopore Technologies (ONT) (version NBE_9065_v109_revY_14Aug2019), as described in the work of Semenzato et al. (2022) [37]. The gDNA of the selected endophyte was sequenced with 11 other non-related gDNA samples. Briefly, 1 µg of each input gDNA was repaired and end-prepped using the NEBNext Companion Module for Oxford Nanopore Technologies Ligation Sequencing (E7180S, New England Biolabs, Ipswich, MA, USA). Upon purification with Agencourt AMPure XP beads (Beckman Coulter, Brea, CA, USA) on a magnetic separator, concentrations of DNA samples were determined using a Qubit 4 Fluorometer and Qubit dsDNA HS Assay Kit (ThermoFisher Scientific). Thus, 500 ng of each end-prepped DNA sample was barcoded using Native Barcoding Expansion 13–24 (EXP-NBD114, ONT) and NEB Blunt/TA Ligase Master Mix (M0367, New England Biolabs). After a purification step, equimolar amounts of barcoded DNA samples were pooled to reach a total of 700 ng and were subjected to adapter ligation. During the subsequent clean-up step, the DNA library was enriched with >3 kb long fragments using the Long Fragment Buffer included in the Ligation Sequencing Kit (SQK-LSK109, ONT). The DNA library was immediately sequenced; therefore, an R9.4.1 Flow Cell (FLO-MIN106D, ONT) was primed with a Flow Cell Priming Kit (EXP-FLP002, ONT). The library was loaded following the instruction provided by the protocol and sequencing was performed with a MinION MK1B (ONT) and the MinKNOW software (22.08.4) for 24 h. Basecalling in a high accuracy mode and demultiplexing were performed using Guppy (6.2.7).

### 2.9. Genome Assembly and Bioinformatic Analysis

*De novo* assembly was accomplished using Canu assembler software, v.2.1.1 [38]. Contig quality was evaluated through QUAST v.5.2.0 [39]. These procedures were performed in a Galaxy environment (https://usegalaxy.eu, accessed on 19 September 2022). The assembled sequence was annotated using the NCBI Prokaryotic Genome Annotation Pipeline (PGAP), v.6.2 (https://www.ncbi.nlm.nih.gov/genome/annotation_prok/, accessed on 21 September 2022). The antiSMASH v.6.1.1 webserver was used for the identification of the gene clusters involved in the biosynthesis of secondary metabolites in the genomes of bacteria [40]. Query genome was uploaded as a FASTA format, and the analysis was performed using a strict method of detection to identify only well-defined clusters that contained genes with significant alignment.

## 3. Results

### 3.1. Electron Microscopy

**Figure 1:**
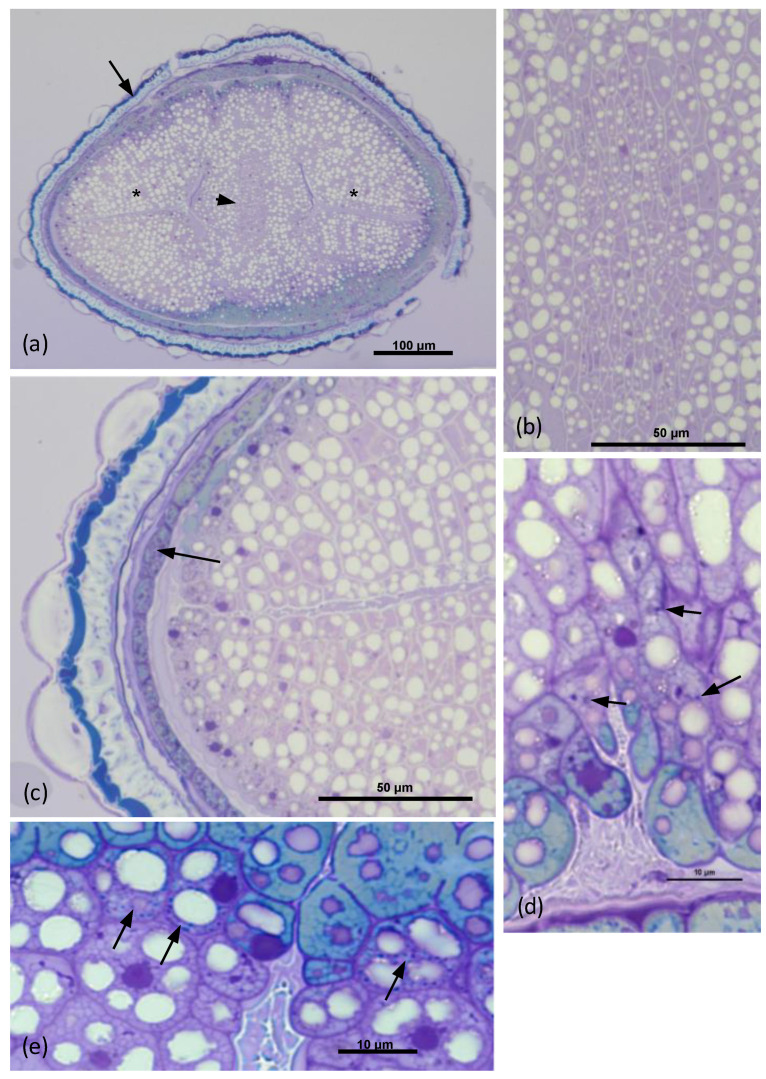
Toluidine blue-stained semithin cross sections of the *O. heracleoticum* seed. (**a**) Cross section approximately in the middle. The central axis of the embryo with the procambium (arrowhead) is in the center of the seed at this point, while the two cotyledons are on the sides (asterisks). The tegument (arrow) has a homogeneous thickness (about 20 μm) around the seed at this point. A more external toluidine blue-positive layer surrounds sclereids. (**b**) Detail of the embryo axis. (**c**) Detail of the tegument zone. The aleurone layer (arrow) is underneath the tegument. (**d**) The cotyledon parenchyma cells close to the aleurone layer contain strongly positive toluidine dots. (**e**) The cells closest to the aleurone layer contain a large number of strongly positive toluidine dots.

By using a light microscope, it can be observed that the seed of *O. heracleoticum* has an oval shape; moreover, a cross section in the middle produced an elliptic 2D image (Figure 1a). The testa had a homogeneous thickness of about 20 μm on average and surrounded the aleurone layer, while in the middle, the masses of the cotyledons filled almost entirely the inside of the seed. The central axis of the embryo with the procambium was in the center of the seed (Figure 1b). In the testa, a tegumental layer was formed by sclereids, while the aleurone layer (arrow) was underneath the most internal part of the integument (Figure 1c). The cotyledon parenchyma cells just beneath the aleurone layer contained strong toluidine-positive dots and white dots (Figure 1d). Some of these cells beneath the aleurone layer contained a particularly high number of strong toluidine-positive dots (Figure 1e).

**Figure 2:**
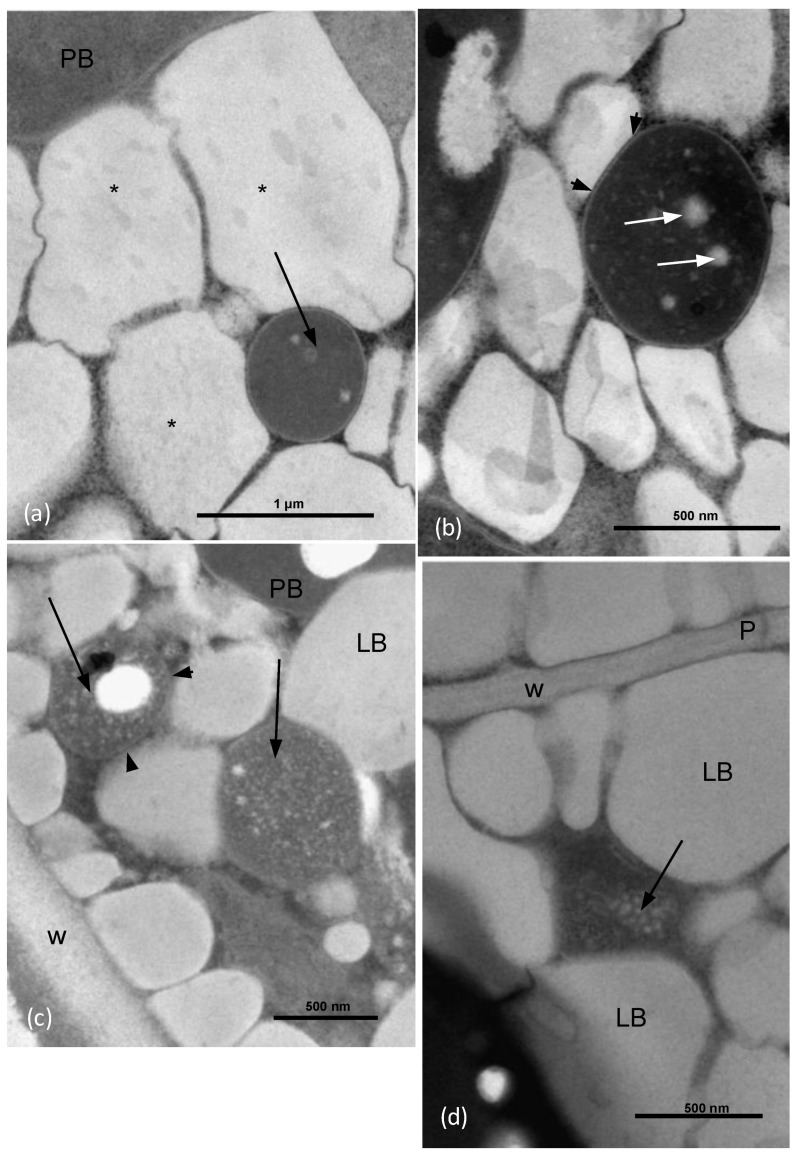
Transmission electron microscope sections of the *O. heracleoticum* seed. Cotyledon parenchyma cells close to the aleurone layer. (**a**) In the parenchyma cells, close to the aleurone layers, some roundish structures (arrow) about 700 nm in diameter on average, with electron-dense content, occupy a space among lipid bodies (asterisks). (**b**) The structures appear to be surrounded by a wall of about 15 nm (arrowheads). Inside, some electron-transparent globules (white arrows) stand out in the electron-dense background. (**c**) Close to the parenchyma cell wall, some roundish structures (arrows) of about 600 nm diameter show granular content and a wall of about 10 nm (arrowheads). (**d**) At some points among the lipid bodies, some elongated structures about 400 nm long, with granular content, did not enter via direct contact with the lipid bodies. No wall is present. LB = lipid body; P = plasmodesmata; PB = protein body; W = cell wall.

Transmission electron microscope (TEM) observations showed that in the parenchyma cells close to the aleurone layers, some roundish structures, about 700 nm in diameter on average, with an electron-dense content, occupied a space among some of the lipid bodies (Figure 2a). These roundish structures were surrounded by a wall about 15 nm thick on average (Figure 2b). Inside these bodies, some electron-transparent globules stood out in the electron-dense background (Figure 2b). Close to the parenchyma cell wall, some roundish structures of an average diameter of about 600 nm showed granular content and a wall of about 10 nm (Figure 2c). In some points among the lipid bodies, some elongated structures about 400 nm long, with granular content, did not enter via direct contact with the lipid bodies, but were surrounded by a granular electron-dense space (Figure 2d). No wall was observed (Figure 2d).

### 3.2. Cultivable Endophytic Bacteria Isolation

The concentration of cultivable endophytes detected inside *O. heracleoticum* seeds ranged between 10^3^ and 10^4^ CFUs/g. A total of 70 endophyte colonies were randomly picked and re-streaked on fresh TSA plates. The isolated endophytes were preliminarily grouped by using phenotypic criteria, such as pigmentation and morphology, and a total of 21 isolates were selected to obtain pure cultures.

### 3.3. Analysis of the Structure of the Seed-Associated Endophytic Community

In order to check the structure of the seed bacterial community at the molecular level, RAPD analysis was performed on all isolates, using the 1253 primer. The profiles obtained were all different (not shown). However, from seven isolates (OR3, OR5, OR6, OR15, OR28, OR56, and OR65), we did not obtain any visible amplification band using primer 1253. For this reason, RAPD analysis was repeated using the AP12 primer, which exhibited a different sequence and different G/C content. The comparison of the fingerprints obtained (not shown) revealed that OR6 and OR15 shared the same RAPD profile. Overall, a total of 20 different RAPD haplotypes were identified in the *O. heracleoticum* seed-associated microbiome, likely corresponding to at least 20 different bacterial strains, assuming that isolates that share the same RAPD fingerprinting correspond to the same bacterial strain [41,42].

### 3.4. Taxonomic Affiliation of the Endophytic Strains

The taxonomic affiliation of the endophytic strains was carried out using the molecular strategy described by Di Cello and Fani (1996) [42]. Accordingly, the 16S DNA from the 20 strains was amplified, as described in the Materials and Methods section. An amplicon of the expected size was obtained from each strain (not shown); each amplicon was subjected to treatment with the restriction endonuclease *Alu*I, which generates species-specific restriction patterns (ARDRA) [43]. In this way, 15 different profiles, as summarized in Table 2, were identified: profile no. 2 was shared between strains OR2 and OR11, profile no. 3 was shared among strains OR3, OR6, and OR15; OR5, OR19, OR28 and OR65 shared profile no. 5; lastly, profile no. 8 was shared between OR8 and OR18.

**Figure 3:**
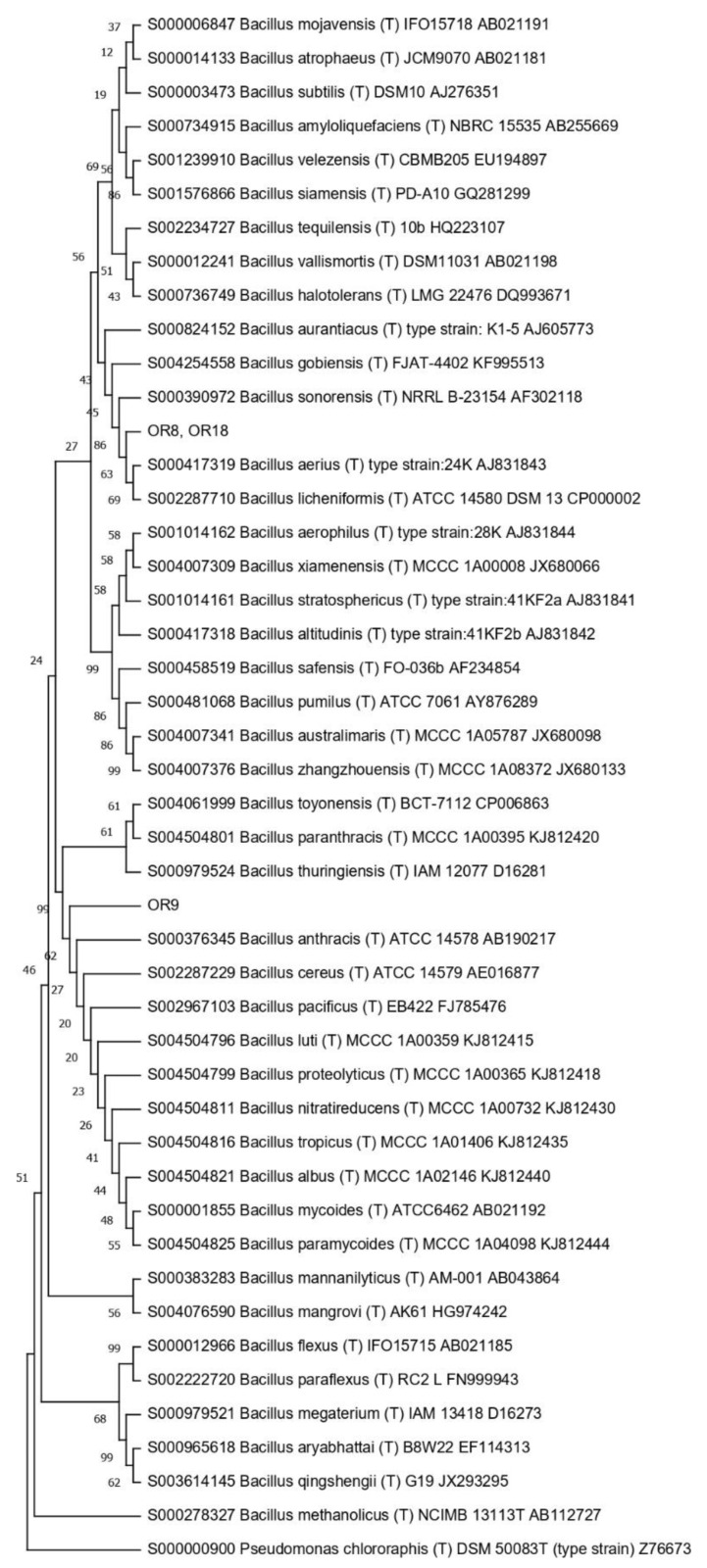
Phylogenetic tree for the genus *Bacillus*.

**Figure 4:**
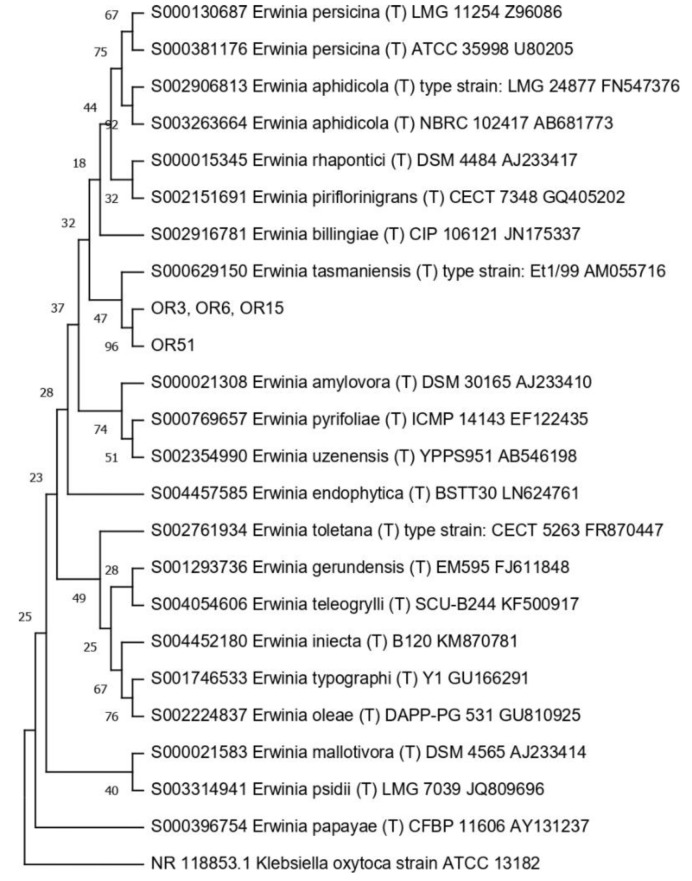
Phylogenetic tree for the genus *Erwinia*.

**Figure 5:**
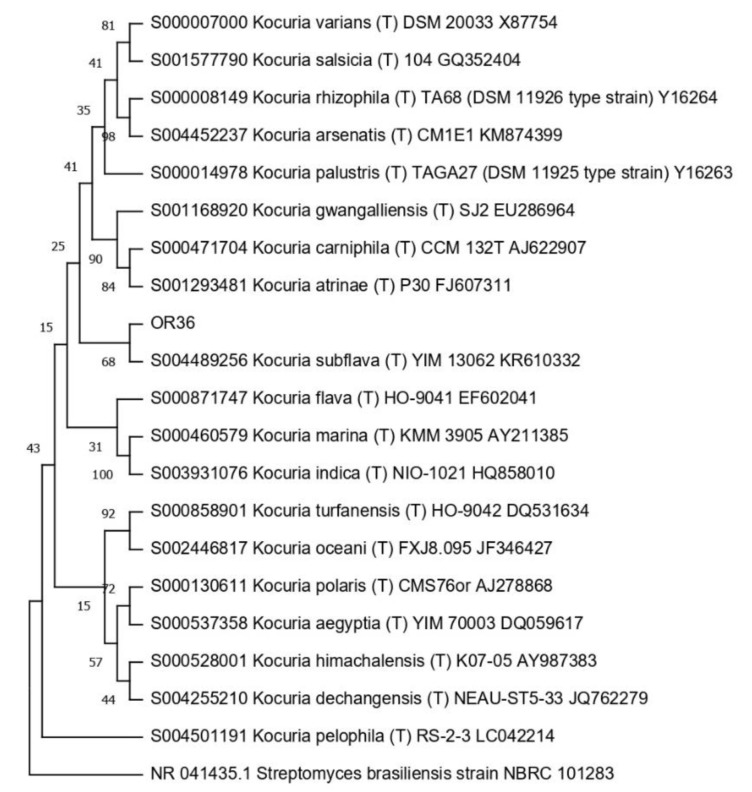
Phylogenetic tree for the genus *Kocuria*.

**Figure 6:**
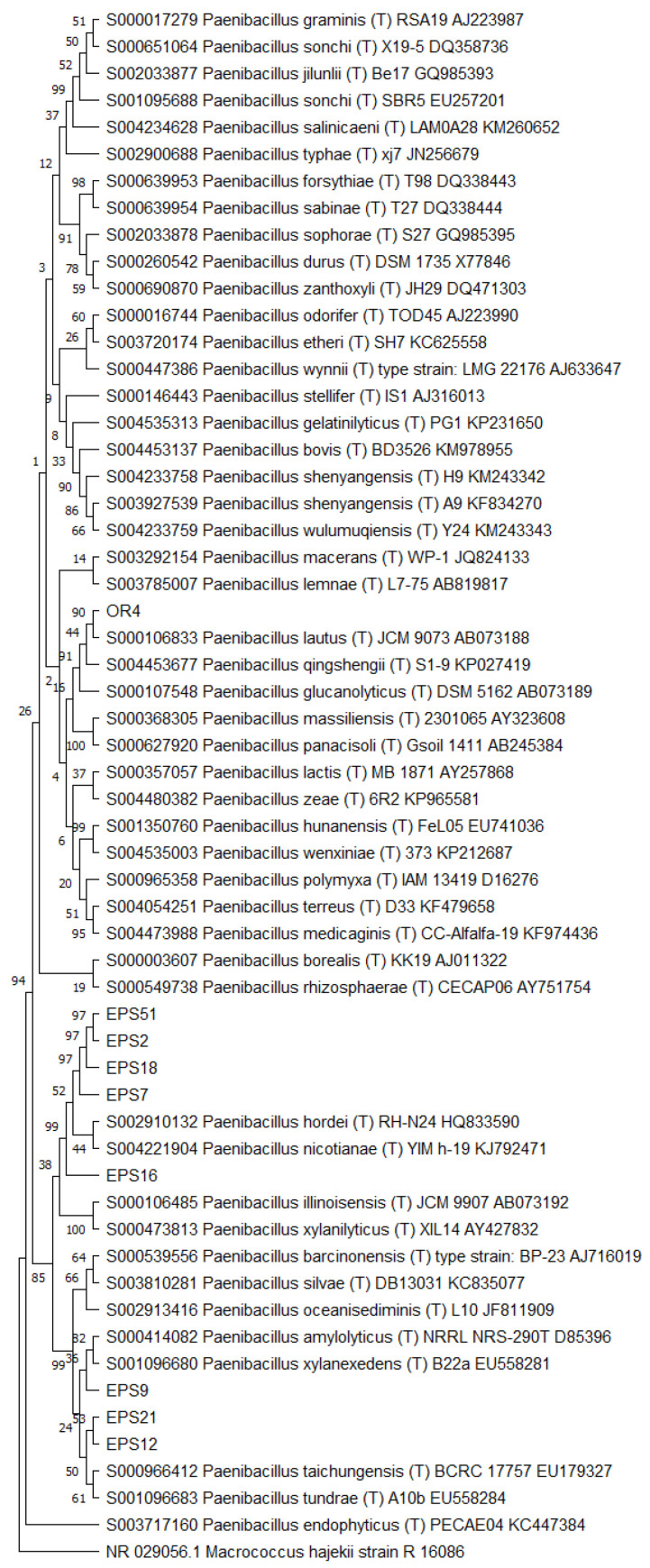
Phylogenetic tree for the genus *Paenibacillus*.

**Figure 7:**
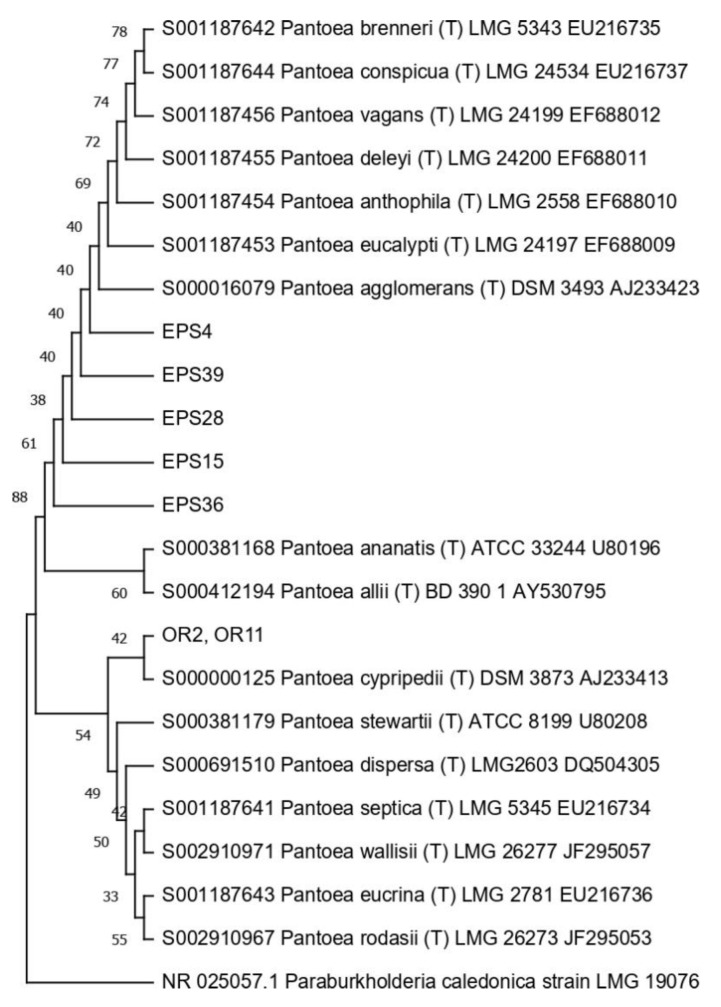
Phylogenetic tree for the genus *Pantoea*.

**Figure 8:**
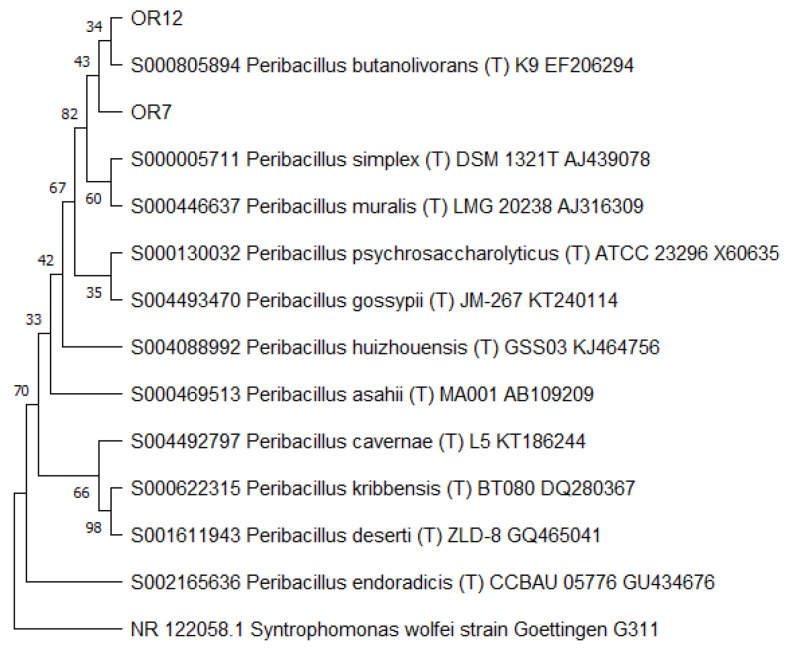
Phylogenetic tree for the genus *Peribacillus*.

**Figure 9:**
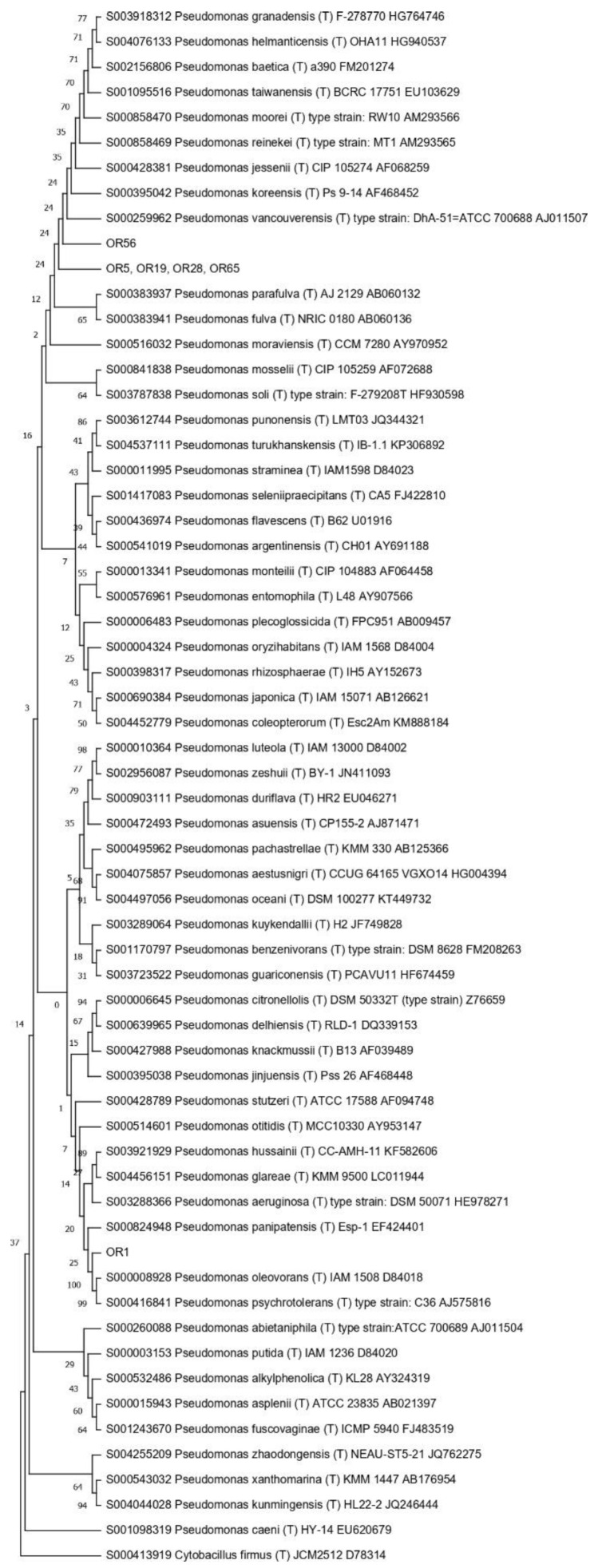
Phylogenetic tree for the genus *Pseudomonas*.

**Figure 10:**
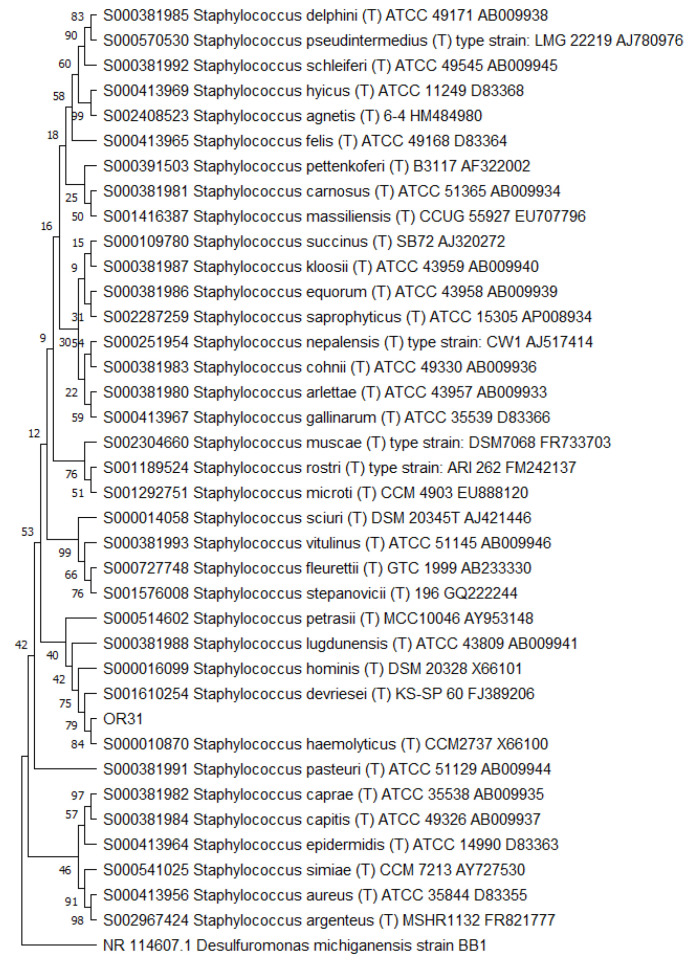
Phylogenetic tree for the genus *Staphylococcus*.

Then, the 16S RNA gene sequence was determined from one representative of each ARDRA group. A total of eight different bacterial genera were identified through 16S gene sequencing and analysis (55.56% Gram-positive and 44.44% Gram-negative). *Pseudomonas* was the most represented genus (28.57%). For each genus, a phylogenetic tree was constructed (Figure 3, Figure 4, Figure 5, Figure 6, Figure 7, Figure 8, Figure 9 and Figure 10), with two of them (i.e., *Paenibacillus* and *Pantoea*) also including the 16S RNA gene sequences from the bacterial endophytes previously isolated from *E. purpurea* seeds [32].

### 3.5. Antibiotic Resistance Profiles

The minimal inhibitory concentration (MIC) of each of the six antibiotics used was evaluated and the relative values are reported in Table 3. Almost all of the endophytic strains were not able to grow in the presence of the lowest concentration of ciprofloxacin (0.5 µg/mL). The bacteria were also reported to be very sensitive to rifampicin; indeed, only 6 out of 20 strains grew in the presence of a concentration of 10 µg/mL of the antibiotic. The resistance profiles obtained for tetracycline and chloramphenicol were variable among the endophytes, which, instead, seemed to tolerate the highest concentrations used for streptomycin and kanamycin (10 and 50 µg/mL). Except for ciprofloxacin, the strains that belong to the genus *Pseudomonas* (in particular, OR5, OR28, OR56 and OR65) turned out to be the most resistant.

### 3.6. Cross-Streaking Test against Burkholderia cepacia Complex Strains

**Figure 11:**
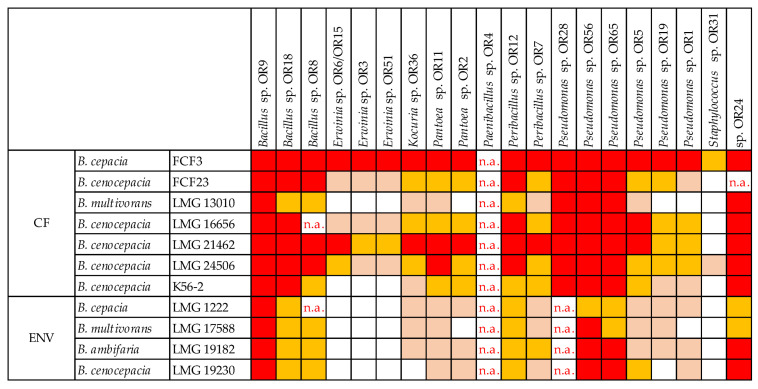
Antagonistic interactions of *O. heracleoticum* seed-associated strains against Bcc strains. The degrees of inhibition observed are indicated as red (complete), orange (strong), salmon (weak) and white (absence of inhibition). n.a. (not available) refers to results that were not obtained.

*O. heracleoticum* seed-associated endophytic strains were tested for the production of antibacterial molecules through the cross-streaking method, as described in the Materials and Methods section, using as targets ten strains that belong to the *Burkholderia cepacia* complex (Bcc), with either clinical or environmental origin. The data obtained are shown in Figure 11.

All tester strains exhibited a complete or strong growth-inhibiting capacity against at least one target strain of human origin. *Bacillus* sp. OR9 showed the strongest antibacterial potential, being able to completely inhibit the growth of all ten targets. Overall, the strains that belong to *Bacillus*, *Peribacillus* and *Pseudomonas* genera were the most effective against both clinical and environmental strains; on the contrary, *Erwinia* and *Staphylococcus* genera showed the weakest antibacterial effect. Finally, the two *B. multivorans* target strains LMG13010, of clinic origin, and LMG17588, of environmental origin, revealed the highest resistance profile, while the *B. cepacia* strain FCF3 was completely inhibited by all the testers, except for *Staphylococcus* sp. OR31.

### 3.7. Genome Sequencing of Bacillus sp. OR9

Based on the cross-streaking test results, *Bacillus* sp. OR9 was chosen to perform whole genome sequencing. The assembled genome of *Bacillus* sp. OR9 has a total length of 6,186,209 bp and embeds 3 contigs (5,512,616 bp, 352,740 bp and 320,853 bp long), with a mean G + C content of 34.93%. The annotation analysis identified a total of 6443 genes, of which 6296 were annotated as coding DNA sequences (CDS), 42 as rRNAs, 100 as tRNAs, and 5 as ncRNA. The genome sequence was deposited in GenBank under the accession number JAOEGP000000000.

The analysis of secondary metabolite biosynthetic gene clusters (BGCs) predicted several types of BGCs, including non-ribosomal peptides (NRPs), terpene, trans-AT polyketide synthase, ribosomally synthesized and post-translationally modified peptides (RiPP-like), betalactone, siderophore, and RiPP recognition elements (RRE-containing) BCGs (Table 4). However, only the one with a percentage equal to or higher than 50% similarity to the clusters available in the database is here described. The *Bacillus* sp. OR9 genome contains a siderophore BCG (from 4,609,931 to 4,624,859 nt) that is accountable for the synthesis of petrobactin. A 100% similarity rate was found for the petrobactin BCG of *Bacillus anthracis str. Ames*.

## 4. Discussion

When considering plant-associated microorganisms, very little attention is paid to the seed microenvironment, even though the seed-associated microbiome may play an important role in germination and seedling development, and also in adult plant growth and fitness stimulation, due to the vertical transmission of a core microbiota [6,13,14,15]. The lack of information about endophytic bacteria is particularly true for medicinal and aromatic plants. To the best of our knowledge, bacterial culturable communities from medicinal plant seeds were studied only in *Echinacea purpurea* [32]; indeed, culture-independent techniques were applied to identify the bacterial and fungal seed-associated core microbiome of different species of the medicinal and aromatic plant *Salvia miltiorrhiza* [22].

In this work, the bacterial endophytic microbiota associated with *Origanum heracleoticum* seeds were observed, isolated, and characterized through a combination of molecular and phenotypic tests. First, we checked the presence of seed endophytes through light and transmission electron microscopy, which revealed that the part of the embryo that was apparently more involved in hosting endophytes was the part closer to the aleurone layer. Through light microscopy, we observed toluidine blue-positive dots in the parenchyma cells, and the pictures taken with the transmission electron microscope showed that the ultrastructure of these dots corresponded to what was already recognized as endophytes by Cardinale et al. (2021), in the seed parenchyma of *E. purpurea* [32]. Also for *O. heracleoticum* seeds the endophytes were endocellular and surrounded by a wall, except for the case of Figure 2d, where the structure may be referrable to a bacterium of the wall-free Mollicutes, which are typical endocellular guests, as pathogens or symbionts, as in the fungus *Geosiphon pyriformis* [44]. The bacterial wall is in strict contact with the host cytoplasm and lipid bodies, suggesting a possible interaction between the endophyte and the host. Pathogenic endocellular bacteria can rarely be visualized in plants, since plant cells tend to undergo a fast death, either due to pathogenicity itself or programmed cell death, as in the case of *Xylella* infection of *Spartium junceum*, where the bacterium can be visualized almost exclusively in the xylem elements [45]. Intracellular bacteria surrounded by a wall can be observed also as a result of phagocytosis in intestinal epithelial cells, as in Figure 2 and Figure 3 in the work of Yamauchi et al. (2000) [46]. In these last images, however, the wall thickness measured was about 60 nm, which is 2–3 times thicker with respect to the endophytes in *O. heracleoticum* seeds. Leaf endophytes in orchid microplants appeared to be devoid of cell wall, as shown in Figure 5 of the article by Esposito-Polesi et al. (2017) [47]. A possible explanation of the different role of the wall may be related to the fact that in the seed, the bacteria and also the plant tissues are quiescent, and the thicker wall may be a result of the genetic programs forming a spore [47].

The cultivable bacterial endophytic fraction was then isolated from surface-sterilized seeds. The concentration of the isolates ranged between 10^3^ and 10^4^ CFUs/g. The same result was obtained for the bacterial endophytes obtained from *Citrus limon* seeds [26], and it is in accordance with previous literature, revealing that endophytic population size can range from 10^1^ to 10^8^ CFUs/g [6,48,49].

Regarding the molecular characterization of the endophytes, the comparative analysis of the 21 haplotypes obtained through the RAPD technique revealed the existence of a high degree of biodiversity at the strain level, in that only two isolates (*Erwinia* sp. OR6 and OR15) shared the same profile. Similar results were obtained when considering the structure of the bacterial endophytic communities isolated from different anatomical compartments of aromatic and medicinal plants [27,50,51].

The degree of biodiversity decreased when considering higher taxonomic levels, i.e., at the genus level. Indeed, the taxonomic analysis performed using the 16S rRNA gene sequences revealed (i) that the bacterial community hosted by the *O. heracleoticum* seeds consists of strains that belong to (at least) eight genera and (ii) the predominance of the strains belonging to the genus *Pseudomonas*. Several endophytic strains appertaining to the *Pseudomonas* genus have been previously identified as common plant growth-promoter bacterium (PGPB), which are able to influence plant growth and development [52]. The phylogenetic analysis also revealed that in most cases, the strains affiliated to the same genus very likely belong to the same species. Two genera (i.e., *Paenibacillus* and *Pantoea*) were shared with *E. purpurea* seed bacterial endophytic communities [32]. However, the analysis of the *Paenibacillus* and *Pantoea* phylogenetic trees reported in Figure 4 and Figure 5, which embed also the 16S rRNA gene sequences from endophytic bacteria isolated from *E. purpurea* seeds, revealed that *E. purpurea* and *O. heracleoticum* seeds host strains that belong to different species of the same genus. This aspect represents an interesting issue, since the two plant species were grown in the same soil (Giardino delle Erbe, Casola Valsenio, Italy) and very close to each other. This finding suggests the existence of a selective force that enables the plant to recall only specific bacteria, i.e., the ones that could best adapt to their inner microenvironment [53].

Among the possible factors believed responsible for the determination of the assembly of endophytic microbiotas, antibiotic resistance profiles might play a crucial role, as demonstrated for different species, but also for different anatomical parts, of *Echinacea* plants [33,53]. Antibiotic resistance could indeed play a role in shaping seed communities’ structure and composition, leading to the selection of those strains exhibiting higher probability of vertical transmission to the next plant generation [32]. Antibiotic resistance tests showed that a great number of the isolates were able to grow in the presence of most of the tested antibiotics and, for some of them, to survive even at higher concentrations. However, the different profiles observed seem to be more likely related to the taxonomic affiliations of the strains (at a genus level), than to their ecological niche. These antibiotic-resistant phenotypes could represent a noteworthy advantage for seed-borne endophytes, ensuring their persistence within seeds, during germination and plant development. On the other hand, these results may raise concerns about the biosafety of those bacteria. Various health risks have been associated with endophytes endowed with plant growth-promoting abilities, especially with some isolates that belong to the genus *Pseudomonas*, but also *Paenibacillus* and *Bacillus* [54]. Thus, hazardous features and antibiotic resistance profiles should be taken into account when considering the biotechnological applications of endophytic strains.

As for antibiotic resistance, the production of bioactive antimicrobial compounds could be another useful feature for seed-associated bacteria establishment inside the seed and for the fitness of the plant’s next generation. It could provide seed-borne bacteria with an advantage in the competition with the soil bacterial communities for the colonization of the seed microenvironment and it could also be involved in the protection of the seedling and the adult plant from biotic stresses [5]. Our results revealed that most of the endophytes isolated from *O. heracleoticum* seeds are able to synthesize antibacterial molecules that are able to inhibit, at different levels, the growth of clinical and environmental strains that belong to the *Burkholderia cepacia* complex (Bcc). Beside plant–microbes interaction implications, these results might be relevant for pharmacological applications. The lack of chemicals able to face the emergence of multidrug-resistant pathogens needs to be addressed, so it is necessary to prioritize the discovery of new natural compounds with biological activity. Bacterial biosynthesis of bioactive molecules with antimicrobial properties has been widely observed and some recent data obtained for some bacterial endophytes isolated from *O. vulgare* L. revealed the potential of endophytes to produce volatile organic compounds that were also found in the essential oil hydrodistilled from the same plant [55]. Exploring the almost untapped natural products produced by bacterial endophytes that reside in medicinal and natural plants could indeed increase the possibilities of finding novel bioactive compounds [16].

To further investigate endophytes’ ability to produce antimicrobial compounds, the genome of *Bacillus* sp. OR9, which showed the highest antibacterial potential against Bcc strains, was sequenced and secondary metabolite biosynthetic gene clusters (BGCs) were searched using the AntiSMASH webserver [40]. Overall, *Bacillus* sp. OR9 genome contains several BGCs accountable for the synthesis of secondary metabolites that could be involved in plant protection against biotic stresses, but also in plant growth promotion. In particular, the siderophore BGC that is responsible for the production of petrobactin was encountered. *Bacillus anthracis str. Ames* holds the same BGC, which plays a role in the virulence of the strain [56]. However, petrobactin production is not exclusive to pathogenic isolates; indeed, numerous innocuous soil-residing isolates are also capable of producing this siderophore [57]. For example, genome analysis of the endophytic strain *Bacillus cereus* T4S, believed to possess plant growth-promoting traits, revealed the presence of the same petrobactin BCG [58]. Thus, it can be hypothesized that this endophytic strain might play a role in encouraging plant growth by means of the acquisition of iron and in protecting its host through the synthesis of secondary metabolites with recognized antibiotic properties [59,60].

## 5. Conclusions

In conclusion, this work is the first characterization of bacterial endophytic culturable microbiota associated with *Origanum heracleoticum* seeds.

*O. heracleoticum* seeds harbor a specific bacterial endophytic community with interesting antimicrobial resistance profiles and antimicrobial properties, which are presumed to facilitate seed microenvironment colonization, transmission to the next generation, and the stimulation of plant growth and defenses. The endophyte production of antimicrobial compounds is a relevant feature that should be further investigated with the hope of demonstrating the pharmacological potential of seed endophytes in facing the antimicrobial resistance issue.

## Figures and Tables

**Table 1:** Target bacterial strains that belong to the *Burkholderia cepacia* complex used in this work.

Strain	Species	Origin
FCF3	*B. cepacia*	CF patients
FCF23	*B. cenocepacia*	
LMG16656	*B. cenocepacia*	
LMG21462	*B. cenocepacia*	
LMG24506	*B. cenocepacia*	
K56–2	*B. cenocepacia*	
LMG13010	*B. multivorans*	
LMG19182	*B. ambifaria*	Environment
LMG1222	*B. cepacia*	
LMG19230	*B. cenocepacia*	
LMG17588	*B. multivorans*	

**Table 2:** Bacterial strains isolated from *O. heracleoticum* seeds.

ARDRA Profiles	RAPD Haplotypes	Isolates	Genus	Accession Number
1	1	OR1	*Pseudomonas*	OP522403
2	2	OR2	*Pantoea*	OP522404
	3	OR11	*Pantoea*	-
3	4	OR3	*Erwinia*	OP522405
	5	OR6; OR15	*Erwinia*	-
4	6	OR4	*Paenibacillus*	OP522406
5	7	OR5	*Pseudomonas*	OP522407
	8	OR19	*Pseudomonas*	-
	9	OR28	*Pseudomonas*	-
	10	OR65	*Pseudomonas*	-
6	11	OR7	*Peribacillus*	OP522408
7	12	OR8	*Bacillus*	OP522409
	13	OR18	*Bacillus*	-
8	14	OR9	*Bacillus*	OP522410
9	15	OR12	*Peribacillus*	OP522411
10	16	OR24	N.A. ^a^	N.A.
11	17	OR31	*Staphylococcus*	OP522412
12	18	OR36	*Kocuria*	OP522413
13	19	OR51	*Erwinia*	OP522414
14	20	OR56	*Pseudomonas*	OP522415

^a^ Not available.

**Table 3:** Minimal inhibitory concentration of the tested antibiotics.

		MIC (µg/mL)					
Genus	Isolate	Str.	Tet.	Cip.	Kan.	Chl.	Rif
*Bacillus*	OR9	50	0.5	0.5	50	2.5	5
*Bacillus*	OR8	50	1.25	0.5	5	25	5
*Bacillus*	OR18	50	1.25	0.5	5	25	5
*N.A. ^a^*	OR24	50	1.25	5	5	5	5
*Erwinia*	OR3	10	12.5	0.5	10	5	25
*Erwinia*	OR6	10	5	0.5	10	5	10
*Erwinia*	OR51	10	12.5	0.5	10	5	25
*Kocuria*	OR36	10	12.5	5	50	5	10
*Paenibacillus*	OR4	n.a. *^a^*	n.a. *^a^*	n.a. *^a^*	n.a. *^a^*	n.a. *^a^*	n.a. *^a^*
*Pantoea*	OR2	10	5	0.5	50	2.5	10
*Pantoea*	OR11	10	2.5	0.5	10	1	10
*Peribacillus*	OR7	50	1.25	0.5	10	25	5
*Peribacillus*	OR12	50	1.25	0.5	5	25	5
*Pseudomonas*	OR1	10	2.5	0.5	2.5	25	5
*Pseudomonas*	OR19	50	1.25	2.5	2.5	5	5
*Pseudomonas*	OR5	>50	25	0.5	10	>50	25
*Pseudomonas*	OR28	>50	25	0.5	10	>50	25
*Pseudomonas*	OR56	>50	25	0.5	10	>50	25
*Pseudomonas*	OR65	>50	>25	0.5	10	>50	25
*Staphylococcus*	OR31	>50	>25	0.5	5	2.5	5

*^a^* Not available.

**Table 4:** Biosynthetic gene clusters identified using AntiSMASH. Nucleotide positions within the genome are reported.

BCG type	From (nt)	To (nt)	Most Similar Known Cluster	% Similarity
Contig 1				
Trans AT-PKS, NRPs	495644	558461	Bacillomycin D	(40%)
Terpene	2943693	2964472	Molybdenum cofactor	(17%)
NRPs	3799358	3842588	-	-
RiPP-like	3864265	3873890	-	-
RiPP-like	3919957	3929781	-	-
Betalactone	3978104	4003065	Fengycin	(40%)
NRPs	4005346	4068767	Puwainaphycin F/ MinutissamideA	(44%)
NRPs-like, NRPs	4190374	4235399	Bacillibactin	(38%)
Siderophore	4609931	4624859	Petrobactin	(100%)
NRPs	4685992	4742532	Octapeptin C4	(17%)
RRE-containing, RiPP-like	5304467	5324736	-	-
Contig 2				
RRE-containing	150714	170974	-	

## Data Availability

16S rRNA gene sequences were submitted to GenBank, under the accession numbers OP522403 to OP522415. The genome sequence of *Bacillus* sp. OR9 was deposited in GenBank under the accession number JAOEGP000000000.
